# Cystic dysplasia of the rete testis accompanying ipsilateral renal agenesis in a young adult: a case report

**DOI:** 10.3325/cmj.2023.64.198

**Published:** 2023-06

**Authors:** Ivan Franin, Igor Grubišić, Lana Postružin Gršić, Monica Stephany Kirigin, Tonći Vodopić, Božo Krušlin

**Affiliations:** 1 Ljudevit Jurak Department of Pathology and Cytology, Sestre Milosrdnice University Hospital Center, Zagreb, Croatia; 2 Department of Urology, Sestre Milosrdnice University Hospital Center, Zagreb, Croatia; 3 Department of Interventional and Diagnostic Radiology, Sestre Milosrdnice University Hospital Center, Zagreb, Croatia; 4 Department of Pathology, Cytology and Forensic Medicine, Varaždin General Hospital, Varaždin, Croatia; 5 Zagreb University School of Medicine, Zagreb, Croatia

## Abstract

A 31-year-old man with left-sided testicular pain lasting a couple of months was referred to our urology department due to a suspected testicular tumor. Physical examination showed a hard, thickened, and small left testis on palpation with a diffuse, inhomogeneous ultrasonographic appearance. After a urologic examination, a left-sided inguinal orchiectomy was performed. The testis, epididymis, and spermatic cord were sent to pathology. Gross examination revealed a cystic cavity filled with brown fluid and the surrounding brownish parenchyma measuring up to 3.5 cm in diameter. Histologic examination showed a cystically dilated rete testis lined with cuboidal epithelium and a positive immunohistochemical reaction to cytokeratins. Microscopically, the cystic cavity was a pseudocyst filled with extravasated erythrocytes and abundant clusters of siderophages. The siderophages extended into the testicular parenchyma, surrounding the seminiferous tubules and spreading out around the ducts of the epididymis, which were also cystically dilated with siderophages inside their lumina. On the basis of clinical data, histological, and immunohistochemical analysis, the patient was diagnosed with cystic dysplasia of the rete testis. The literature shows an association between cystic dysplasia of the rete testis and ipsilateral genitourinary anomalies. Therefore, our patient underwent a multi-slice computed tomography scan, which revealed ipsilateral renal agenesis, a right seminal vesicle cyst reaching up to the iliac arteries, and a multicystic formation cranial to the prostate.

Cystic dysplasia of the rete testis is a rare, benign cause of testicular mass, mostly observed in children. Around 60 cases have been recorded since the initial description in 1973 (1). Cystic dysplasia of the rete testis is characterized by irregular cystic areas lined by cuboidal epithelium in the mediastinum or rete testes. It frequently coexists with genitourinary tract abnormalities, most prominently renal agenesis (2). Only two adult patients, aged 23 and 63, have been reported in the literature so far.

## Case report

**Figure 1:**
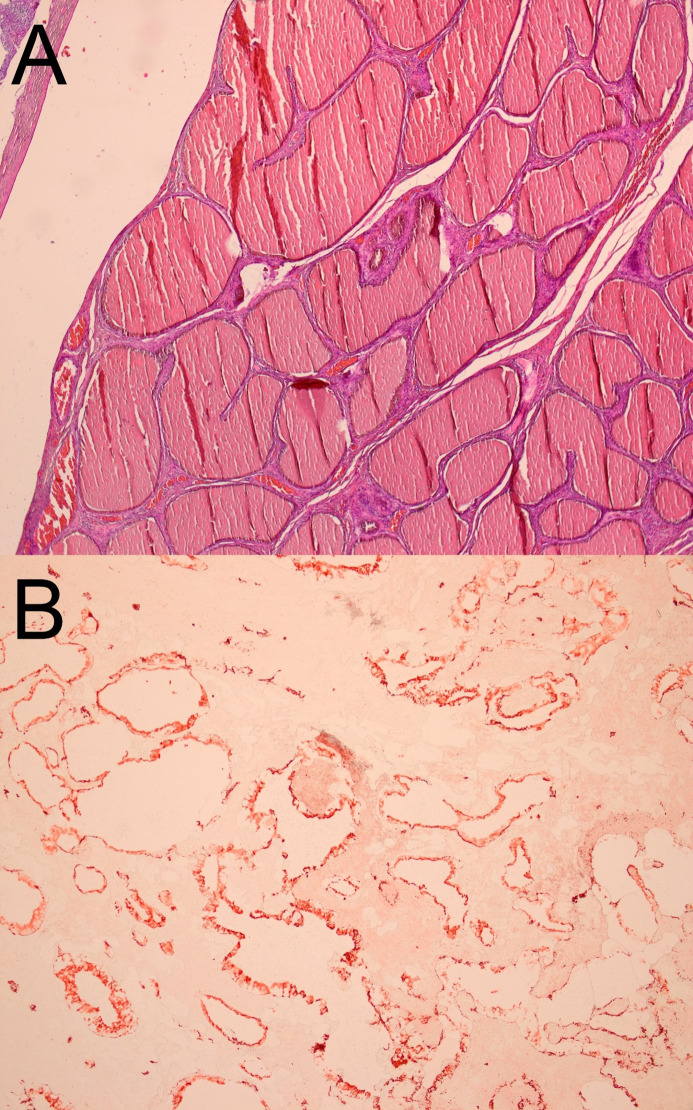
**(A)** Hematoxylin and eosin staining showing a dilated rete testis. (**B)** Cytokeratin staining showing a dilated rete testis.

**Figure 2:**
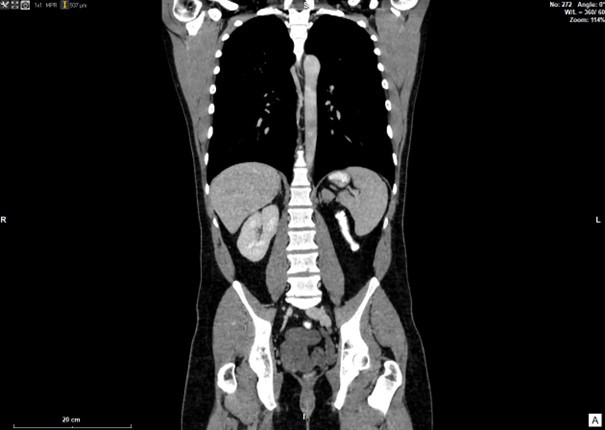
Multi-slice computed tomography with gastrografin showing renal agenesis and a multicystically dilated seminal vesicle.

A 31-year-old male patient was referred to our Urology Department from an outside institution. He had been experiencing left-sided testicular pain for a few months and was suspected of having a testicular tumor. On palpation, the left testis was firm, thickened, and small. Ultrasonography showed a widespread, inhomogeneous appearance. A left-sided inguinal orchiectomy was performed. The spermatic cord, epididymis, and testis were sent to pathology. Gross inspection revealed a 3.5 cm-diameter cystic cavity with brown fluid content and the surrounding brownish parenchyma. Histologic examination showed a cystically dilated rete testis lined with cuboidal epithelium, positive for cytokeratins. Microscopically, the cystic cavity contained extravasated erythrocytes and large clusters of siderophages. The ducts of the epididymis were cystically dilated and had siderophages inside their lumina. Seminiferous tubules of the testicular parenchyma were also surrounded by siderophages (Figure 1 A, B). The diagnosis of cystic dysplasia of the rete testis was based on clinical information, histological examination, and immunohistochemical analysis. The relationship between ipsilateral genitourinary abnormalities and cystic dysplasia of the rete testis is well-known. Therefore, the patient underwent a multi-slice computed tomography scan, which revealed ipsilateral renal agenesis, a right seminal vesicle cyst reaching up to the iliac arteries, and a multicystic formation cranial to the prostate (Figure 2). The patient fully recovered after surgery and was doing well during follow-up. The timeline of diagnostic tests and interventions is shown in Table 1.

**Table 1:** The timeline of diagnostic tests and interventions

Date	Diagnostic tests and interventions
March 8, 2021	Urological examination
March 9, 2021	Orchiectomy
March 25, 2021	Pathology report results
March 26, 2021	Multi-slice computed tomography

## Discussion

The precise etiology of cystic dysplasia of the rete testis is still unknown. One theory is that the mediastinum testis gradually degenerates into small cysts due to a lack of connection between the mesonephric duct and the germinal epithelium at the level of the rete testis (1). This theory may also explain the associated urinary tract problems. The ureteral bud, from which the mesonephric duct develops, eventually gives rise to the kidney. Another theory involves excessive fluid secretion in immature seminiferous tubules without a lumen (3). Gradual canalization of the tubules during childhood may cause the cysts' spontaneous regression (4).

The age at presentation of cystic dysplasia of the rete testis ranges from birth to 18 years, with a median of 5.2 years. Only two adult patients, aged 23 and 63 years, have been reported in the literature but were excluded from the review by Contini et al (2) due to the age limit. Our patient is the second oldest patient according to the age at presentation reported in the English-language literature. He presented with testicular pain, as was the case with 9% of the reported patients. The urogenital system anomaly most commonly associated with cystic dysplasia of the rete testis is ipsilateral renal agenesis (50%), which was also observed in our patient. Alongside ipsilateral renal agenesis, we observed enlarged seminal vesicles, present in 4.5% of other patients. Overall, 48.5% of other patients underwent orchiectomy (2). Surgery should only be used in the case of an ambiguous diagnosis (5). Patients who fulfil the criteria for cystic dysplasia of the rete testis should undergo yearly scrotal ultrasonography (5). When non-surgical therapy is chosen, close monitoring during the initial several months after diagnosis is essential. However, a definite diagnosis and the exclusion of a malignant cystic testicular lesion require surgical biopsy and histological confirmation, particularly when the lesion does not regress on ultrasound (2). To conclude, because of the late presentation of this lesion in our patient, we suggest considering cystic dysplasia of the rete testis as a possible differential diagnosis in adults.
